# Rheuma nach COVID-19-Infektion oder Impfung

**DOI:** 10.1007/s41970-023-00220-5

**Published:** 2023-02-27

**Authors:** Manfred Herold

**Affiliations:** Universitätsklinik für Innere Medizin II, Rheuma- und Infektionslabor, University of Innsbruck and Medical University of Innsbruck, Anichstr. 35, 6020 Innsbruck, Österreich

**Keywords:** SARS-CoV‑2, Arthralgie, Arthritis, Myositis, Tendinitis, SARS-CoV‑2, Arthralgia, Arthritis, Myositis, Tendinitis

## Abstract

Sowohl nach COVID-Infektion als auch nach ein oder mehreren COVID-Impfungen können rheumatische Beschwerden beginnen. In beiden Fällen scheint der Mechanismus ähnlich zu sein und mit dem Coronavirus oder seinen spezifischen Folgen zusammenzuhängen. Zumeist wird von einer reaktiven Arthritis gesprochen, wenngleich die Bezeichnung COVID-19-assoziierte Arthritis für das Beschwerdebild eher zutreffen dürfte. In Relation zur Zahl der COVID-Infizierten und der COVID-geimpften ist die Zahl der Fälle, in denen es zu COVID-assoziierten Beschwerden kommt, außerordentlich gering und die Prognose scheint eher gut zu sein.

## Abstract (en)

Rheumatic complaints can begin both after COVID infection and after one or more COVID vaccinations. In both cases, the mechanism seems to be similar and related to the coronavirus or its specific consequences. In most cases it is named reactive arthritis although the term COVID-19-associated arthritis is more likely to apply to the symptoms. In relation to the number of COVID-infected and COVID-vaccinated people, the number of cases of COVID-related symptoms is extremely low and the prognosis seems to be rather good.

Seit Beginn der Pandemie Ende 2019 mehren sich Berichte, dass nach COVID-Infektion chronische entzündlich-rheumatische Erkrankungen beginnen können. Rheumatische Beschwerden im Zusammenhang mit viralen oder bakteriellen Infekten sind nicht ungewöhnlich, können auch einige Tage über die Infektion anhalten und sind üblicherweise selbstlimitierend. Demgegenüber stehen Beschwerden, die Tage bis Wochen nach einem Infekt beginnen, über Wochen bis Monate anhalten und sich mitunter zur chronischen entzündlich-rheumatischen Erkrankung entwickeln.

Nach Abklingen einer akuten Infektion mit SARS-CoV‑2 können Krankheitssymptome noch über längere Zeit persistieren und werden unter dem Begriff Long COVID zusammengefasst. Die häufigsten Beschwerden sind andauernde Müdigkeit (Fatigue), anhaltendes Krankheitsgefühl („post-exertional malaise“, PEM) mit eingeschränkter körperlicher und geistiger Leistungsfähigkeit, Kurzatmigkeit, Geruchs- und Geschmacksstörungen und andere Symptome wie Gelenk- und Muskelschmerzen (https://www.sozialministerium.at/Corona/allgemeine-informationen/long-covid.html). Schätzungen zufolge leiden etwa 10 % der COVID-19-Überlebenden an Long COVID [1, 2]. Unabhängig von Patienten mit Long COVID wird aber zunehmend von Patienten berichtet, bei denen nach einer COVID-19-Infektion Symptome einer chronischen entzündlich-rheumatischen Erkrankung beginnen [3–5]. Dazu zählen Symptome einer Arthritis, Myositis, autoinflammatorische Symptome, Beschwerden passend zu Kollagenosen oder chronischen entzündlich-rheumatischen Erkrankungen, die nicht assoziiert sind mit Autoantikörpern, wie zum Beispiel Psoriasis mit Psoriasisarthritis oder andere Spondylarthropathien [6, 7].

## Reaktive Arthritis oder virusassoziierte Arthritis?

Eine Arthritis, die Tage bis Wochen nach einem dokumentierten Infekt auftritt, wird üblicherweise als reaktive Arthritis (ReA) bezeichnet. Ausgehend von der Beobachtung des deutschen Militärarztes Hans Reiter, dem während des 1. Weltkriegs auffiel, dass Harnwegsinfekt, Augenentzündung und Entzündung der Gelenke mitunter begleitet von typischen Hauterscheinungen (Reiter-Dermatose) häufig gemeinsam auftreten. Bis vor wenigen Jahren wurde bei Vorliegen von zumindest 3 der 4 Symptome der Begriff Reiter-Syndrom oder Reiter-Trias (Urethritis, Konjunktivitis, Arthritis) verwendet. Die Bezeichnung ReA wurde 1963 eingeführt, 1983 vorläufige Diagnosekriterien formuliert und 1991 die ReA als Untergruppe den Spondylarthritiden zugeordnet [8]. Seit 2000 wurde wegen der nationalsozialistischen Zugehörigkeit von Dr. Hans Reiter empfohlen, den Begriff Reiter-Syndrom nicht mehr zu verwenden.

Arthritiden, die nach bekannten Infektionen auftreten, können in Abhängigkeit vom Nachweis im Gelenkpunktat in 3 Gruppen eingeteilt werden. Eine infektiöse Arthritis liegt vor, wenn im Gelenkpunktat ein Erreger nachgewiesen werden kann, eine postinfektiöse Arthritis bei Nachweis von mikrobiellen Bestandteilen, aber Fehlen eines intakten Erregers, und eine ReA bei eindeutiger Anamnese, aber Fehlen von Erregern oder Erregerbestandteilen (Tab. 1).

**Table 1:** 

Gruppe	Infektion bekannt	Erreger im Gelenk	Erregerprodukte im Gelenk	Form der Arthritis
1	Ja	+	+	Infektiös
2	Ja	–	+	Postinfektiös
3	Ja	–	–	Reaktiv

Bezüglich der Bezeichnung einer Arthritis nach COVID-19-Infektion besteht noch kein Konsens. In den meisten Arbeiten wird von einer ReA nach COVID-19-Infektion berichtet [9–13]. Die ReA wird derzeit zu den Spondylarthritiden gezählt, im internationalen Konsens formulierte Klassifikations- oder Diagnosekriterien liegen aber noch nicht vor.

Die Richtigkeit der Bezeichnung ReA im Zusammenhang mit SARS-CoV‑2 wird aber immer wieder hinterfragt und diskutiert, ob anstelle von ReA die Bezeichnung virale Arthritis, SARS-CoV‑2 assoziierte Arthritis oder Post-COVID-Arthritis nicht eher zutreffen würde [14–18]. Aus den zahlreichen Fallberichten über das Auftreten einer akuten Arthritis nach COVID-Infektion zeichnet sich zusammenfassend ein Krankheitsbild ab, das sich in einigen Punkten doch von einer klassischen ReA unterscheidet (Tab. 2).

**Table 2:** 

	Klassische ReA	Post-COVID-Arthritis
Alter (Jahre)	Vorwiegend unter 40	Vorwiegend über 45
Geschlechtsverteilung	Männer überwiegen (m:w ≈ 6:1)	Etwa gleich verteilt
Vorangehendes Ereignis	Darm- oder Urogenitalinfekt	Atemwegsinfekt
Auslöser	Bakterium	Virus
Phänotyp	Spondylarthritis	Verschiedenartig
HLA-B27	Überwiegend positiv (bis ~ 80 %)	–
Bevorzugte Gelenke	Große Gelenke	Kleine Gelenke
Chronizität	Ca. 30 % chronisch (> 3 Monate)	Heilung innerhalb 3 Monate
Behandlung	Wie Spondylarthritiden	Niederdosiert Steroid ± NSAR

Die reaktive Arthritis zeigt sich typischerweise als asymmetrische Oligoarthritis mit Dominanz der großen Gelenke. Die Post-COVID-Arthritis neigt eher zu diffuser Verteilung mit Befall von Hand- und Sprunggelenken sowie Finger- und Zehengelenken. Die ReA findet man eher bei jüngeren Patienten, eine Post-COVID-Arthritis vermehrt bei Patienten in der zweiten Lebenshälfte. Typischer Auslöser einer ReA sind Bakterien, bei Post-COVID Viren. Eine ReA wird häufiger bei Männern gesehen, eine Post-COVID-Arthritis scheint in beiden Geschlechtern etwa gleich verteilt zu sein. Patienten mit ReA sind überwiegend HLA-B27-positiv, bei Patienten mit einer Post-COVID-Arthritis ist eine Anfälligkeit von HLA-B27-positiven Patienten bisher noch nicht beobachtet worden [11, 16]. In einer Zusammenfassung von 26 Patienten mit COVID-assoziierter Arthritis, die 1 Woche oder später nach COVID-Infektion aufgetreten ist, wurde auch der HLA-Status beschrieben, soweit er vorhanden war. Von 26 angeführten Patienten waren 3 positiv für HLA-B27, 12 negativ und bei 11 Patienten war der HLA-Status nicht bekannt [17]. Ähnliche Ergebnisse werden auch in einer zusammenfassenden Arbeit über 20 Patienten angeführt, von denen bei 10 Patienten der HLA-B27-Status bekannt war, 2 Patienten waren positiv, 8 negativ [10]. In einer weiteren Studie waren von 13 Patienten mit Post-COVID-Arthritis, die auch auf ihren HLA-B27-Status überprüft wurden, 5 positiv [13]. Auch in Einzelfallberichten, in denen der HLA-Status erwähnt wird, überwiegt der HLA-B27-negative Befund [9, 11].

Das klinische Bild einer ReA wurde auch bei anderen Viruserkrankungen [10] wie HIV („human immunodeficiency virus“), Hepatitis B und C [19, 20], Epstein-Barr (EBV), Parvovirus, Dengue, Chikungunya [21], Zika beschrieben. Ob das Virus selbst oder ein begleitender bakterieller Infekt die ReA auslöst, ist nicht geklärt. Im Falle der beschriebenen Post-COVID-Arthritiden ist allen beschriebenen Fällen gemeinsam die kurz zurückliegende und durch Antigentest nachgewiesene COVID-Infektion.

## Arthritis nach COVID-Infektion

Das Auftreten einer Arthritis nach einer COVID-Infektion wird in zahlreichen Publikationen und Fallberichten belegt. Eine Suche in PubMed mit den Begriffen „arthritis“ und „COVID“ ergab 1958 Treffer.

Eine Literatursuche von Dezember 2019 bis Dezember 2021 nach klar definierten Suchkriterien ergab 68 Artikel, aus denen nach entsprechenden Selektionen 25 Fälle aus 22 Artikeln extrahiert und zusammengefasst werden konnten [13]. Von den 25 Patienten waren 11 Frauen und 14 Männer im Alter von 45 Jahren (Mittelwert). Die Zeit von Infektion und Auftreten der Arthritis war zwischen 6 und 48 Tagen. In 20 Fällen wurde peripherer Gelenkbefall beschrieben. Betroffen waren Knie (*n* = 11), Fuß (*n* = 2), Sprunggelenke (*n* = 9), Hüfte (*n* = 3), Handgelenke (*n* = 6), Hände (*n* = 3), Ellenbogen (*n* = 3) und Schultern (*n* = 4). Vereinzelt wurde über Tendinitis und Daktylitis berichtet [13].

Eine weitere Übersichtsarbeit, in der eine Fallbeschreibung ergänzt wird durch einen komprimierten Literaturüberblick bis Juli 2021, ergibt nicht unerwartet ähnliche Ergebnisse. Die Dauer von der Diagnose COVID bis zur Diagnose ReA war 7–90 Tage (Median 18). Die am meisten betroffenen Gelenke waren Knie, Sprunggelenke und Interphalangealgelenke [22].

In einem zusammenfassenden Bericht über 23 eigene Patienten und 21 Patienten aus vorangehenden Berichten war die durchschnittliche Zeit von Beginn der COVID-Symptome bis zum Beginn einer Arthritis 23 Tage (Mittelwert). Die am häufigsten befallenen Gelenke waren Knie, Sprunggelenke und Interphalangealgelenke [18].

Die Prognose der Arthritis nach COVID scheint gut zu sein. Die Therapie war in den meisten Fällen rein symptomatisch mit NSAR und Steroid lokal oder systemisch. Basistherapeutika wie Salazopyrin, Hydroxychloroquin, Methotrexat oder Biologika wurden nur selten verabreicht.

## Andere rheumatische Beschwerden nach COVID-Infektion

Eine COVID-Infektion scheint auch Autoimmunerkrankungen zu verursachen. Berichtet wurde über Guillain-Barré-Syndrom (GBS), autoimmun hämolytische Anämie, Antiphospholipidsyndrome (APS), systemischen Lupus erythematodes (SLE), multiple Sklerose (MS), akute disseminierte Encephalomyelitis, Multisystem-Entzündungssyndrom bei Kindern („multisystem inflammatory syndrome in children“, MIS-C) und rheumatoide Arthritis (RA) [3]. Vermutet wird eine COVID-bedingte Störung des immunologischen Gleichgewichts mit Förderung von Autoimmunreaktionen einschließlich Autoantikörperbildung. Es handelt sich durchweg um einzelne beschriebene Fälle. Über eine größere Ansammlung von Autoimmunerkrankungen nach COVID-Infektion hat bisher kein Zentrum berichtet.

Einige Studien vermuten, dass Patienten mit Autoimmunerkrankungen vermehrt zu Hospitalisierung, schwereren Komplikationen und höherer Mortalität durch COVID neigen. Dieser Vermutung liegt die Überlegung zugrunde, dass Patienten mit Autoimmunerkrankungen im Vergleich zur gesunden Normalbevölkerung empfänglicher sind für eine SARS-CoV-2-Infektion als Folge des geschwächten Immunsystems, der Anwendung von immunmodulierenden Medikamenten und der bereits eingetretenen Organschäden. Demgegenüber lassen andere Studien vermuten, dass die regelmäßige Medikamenteneinnahme Patienten mit Autoimmunerkrankungen vor schweren COVID-Komplikationen schützt [3].

Neben der am häufigsten beschriebenen COVID-assoziierten ReA werden auch andere chronische entzündlich-rheumatische Erkrankungen als COVID-bedingt vermutet und beschrieben. Berichtet wurde über den Beginn unterschiedlicher Formen einer Vasculitis nach COVID-Infektion [23, 24], wobei auch COVID-assoziierte Vaskulitis bei Kindern und Jugendlichen beschrieben wurde [25, 26].

Nicht nur über vereinzelt nach COVID begonnene chronische entzündlich-rheumatische Erkrankungen, die allgemein als Autoimmunerkrankung gelten, wird berichtet, sondern auch über autoinflammatorische Syndrome wie die systemische juvenile idiopathische Arthritis (alte Bezeichnung: juveniler Morbus Still) des Kindes [27, 28] sowie Morbus Still des Erwachsenen (AOSD, „adult onset still syndrome“) [29, 30] oder über Morbus Behçet [31].

Seit Beginn der Pandemie bestand die Sorge, dass Patienten mit Autoimmunerkrankung in Bezug auf COVID verstärkt gefährdet seien und ein höheres Risiko für schwerwiegende Komplikationen hätten. Die bisherige Erfahrung hat das aber nicht wirklich bestätigt [32–34].

## Rheumatische Beschwerden nach COVID-19-Impfung

Die Impfskepsis ist hoch unter der Allgemeinbevölkerung und unter Patienten mit chronischen entzündlich-rheumatischen Erkrankungen. Die internationalen rheumatologischen Gesellschaften haben von Anfang an empfohlen, die Impfung gegen SARS-CoV‑2 anzunehmen, da sie den besten Schutz bietet vor schweren oder fatalen Verläufen [35–37]. Bisher gibt es keinen Hinweis, dass Patienten mit chronischen entzündlich-rheumatischen Erkrankungen vermehrt zu Impfreaktionen neigen oder durch die Impfung eine Reaktivierung ihrer Grundkrankheit ausgelöst werden könnte [38]. Dennoch scheint in Einzelfällen eine chronische entzündliche oder autoinflammatorische Erkrankung durch eine COVID-Impfung induziert zu werden [39].

Nach COVID-19-Impfung kann es zu ähnlichen rheumatischen Krankheitsbildern kommen wie nach COVID-Infektion [40–44]. Beschrieben wurden nach SARS-CoV‑2 Impfung unter anderem der Beginn einer diffus kutanen Sklerose, ANCA-assoziierte Vaskulitis [45], RS3PE(„remitting seronegative symmetrical synovitis with pitting edema“)-Syndrom [46], Still-Syndrom des Erwachsenen (AOSD) [47] und entzündliche Muskelerkrankungen wie Polymyalgia rheumatica (PMR) oder PMR-ähnlichen Symptomen [48], Myositis [49] und Myokarditis [50].

Trotz der zunehmenden Berichte über Autoimmunsyndrome nach COVID-Impfung ist die Gesamtzahl in Bezug auf die Zahl der Geimpften sehr gering und spricht nicht gegen die Vorteile einer Impfung.

## Diskussion

Vereinzelt reagieren Patienten nach COVID-Infektion oder Impfung mit dem akuten Symptomen passend zu einer chronisch-autoimmunen oder chronisch-inflammatorischen Erkrankung. Der Zusammenhang zwischen COVID und rheumatischen oder muskuloskelettalen Beschwerden oder Erkrankungen ist unklar [5]. Auch bei anderen Viruserkrankungen wurde ein Zusammenhang mit akuter oder chronischer Arthritis beschrieben [3]. SARS-CoV‑2 und Impfung scheinen Autoimmunität und Autoinflammation über ähnliche Mechanismen zu induzieren. Die beobachteten Erkrankungen sind ähnlich nach Infektion und Impfung.

## Fazit für die Praxis

Bei neu einsetzenden Symptomen einer autoimmunbedingten oder autoinflammatorischen Erkrankung sollte man auch an eine Reaktion nach COVID-Infektion oder -Impfung denken. Die Prognose ist eher gut.
